# ATP12A promotes mucus dysfunction during Type 2 airway inflammation

**DOI:** 10.1038/s41598-018-20444-8

**Published:** 2018-02-01

**Authors:** Alison T. Lennox, Stefanie L. Coburn, John A. Leech, Elisa M. Heidrich, Thomas R. Kleyman, Sally E. Wenzel, Joseph M. Pilewski, Timothy E. Corcoran, Mike M. Myerburg

**Affiliations:** 1 University of Pittsburgh, Department of Medicine, Division of Pulmonary, Allergy, and Critical Care Medicine, UPMC Montefiore Hospital, NW 628, 3459 Fifth Ave, Pittsburgh, PA 15122 USA; 2 University of Pittsburgh, Department of Medicine, Renal-Electrolyte Division, UPMC Montefiore Hospital, NW 628, 3459 Fifth Ave, Pittsburgh, PA 15122 USA; 3 Department of Cell Biology, Department of Pharmacology and Chemical Biology, University of Pittsburgh, Pittsburgh, PA 15122 USA; 4 Pulmonary Medicine, Allergy, and Immunology, Children’s Hospital of Pittsburgh, Pittsburgh, PA 15122 USA; 5 Department of Chemical and Petroleum Engineering, Department of Bioengineering, University of Pittsburgh, Pittsburgh, PA 15122 USA

## Abstract

Allergic airway disease is known to cause significant morbidity due to impaired mucociliary clearance, however the mechanism that leads to the mucus dysfunction is not entirely understood. Interleukin 13 (IL-13), a key mediator of Type 2 (T2) inflammation, profoundly alters the ion transport properties of airway epithelium. However, these electrophysiological changes cannot explain the thick, tenacious airway mucus that characterizes the clinical phenotype. Here we report that IL-13 dramatically increases the airway surface liquid (ASL) viscosity in cultured primary human bronchial epithelial cells and thereby inhibits mucus clearance. These detrimental rheological changes require ATP12A, a non-gastric H^+^/K^+^-ATPase that secretes protons into the ASL. ATP12A knockdown or inhibition prevented the IL-13 dependent increase in ASL viscosity but did not alter the ASL pH. We propose that ATP12A promotes airway mucus dysfunction in individuals with T2 inflammatory airway diseases and that ATP12A may be a novel therapeutic target to improve mucus clearance.

## Introduction

Mucociliary clearance (MCC) is a primary innate defense mechanism of the conducting airways, enabling inhaled particulate matter and pathogens to be expelled^1^. Accumulating evidence indicates that mucus clearance is dependent on a thin layer of fluid, known as the airway surface liquid (ASL), which acts as a low-viscosity medium to facilitate ciliary beat and allows mucus to glide along the luminal surface^2–7^. The ASL volume is maintained by the osmotic driving force established by the transport of salt across the airway epithelium. In Cystic Fibrosis (CF), reduced transepithelial chloride (Cl^−^) and bicarbonate (HCO_3_^−^) ion transport through the dysfunctional cystic fibrosis transmembrane conductance regulator (CFTR) renders the airway vulnerable to dehydration and reduces the ASL pH. The dehydration and acidification of the ASL impairs MCC and innate immunity leading to airway obstruction, inflammation, chronic infection, and ultimately, premature respiratory failure^4–12^.

Patients with allergic airway diseases characterized by elevated levels of Type 2 (T2) inflammation, such as eosinophilic asthma, allergic bronchopulmonary aspergillosis (ABPA), and allergic rhinitis, have several clinical features suggestive of dysfunctional mucus clearance. Mucus plugging is a common pathological feature in these diseases and leads to small airways obstruction, subsegmental lung collapse, and can precipitate respiratory failure. Furthermore, asphyxiation is often the defining cause of death in cases of fatal asthma, where “extensive obstruction of the conducting airway by tenacious mucus exudates” is described on pathological examination^13–16^. Despite these significant findings, the mucus dysfunction in allergic airway disease is often underappreciated by clinicians, perhaps because small airway mucus infrequently results in a productive cough. Moreover, targeted therapies to treat the muco-occlusive disease associated with T2 inflammation are not currently available^17^.

In contrast to the pathophysiology of CF, the airway epithelium exhibit increased electrogenic Cl^−^ secretion and decreased electrogenic sodium (Na^+^) absorption during T2 inflammation. This has led to the belief that the asthmatic airway is highly secretory, and this hypothesis has been previously supported by the finding that the ASL height was increased when bronchial epithelial (HBE) cells were exposed to the T2 inflammatory cytokine, interleukin-13 (IL-13)^17–22^. However, the “rubbery” secretions found in asthmatic airways are dehydrated with a highly dense elastic modulus^23–25^. These biophysical properties are inconsistent with well-hydrated mucus, which would be highly deformable and loosely packed. Thus, the mucus dysfunction seen in T2 inflammatory airway disease is not explained by the canonical mediators of airway ion transport.

The predilection towards mucus obstruction in T2 airway disease has been attributed to excessive mucus production and alteration of the relative composition of the airway mucins. Bonser *et al*. recently reported that MUC5AC is upregulated and becomes membrane tethered in response to IL-13 and that membrane tethered MUC5AC impairs MCC and may promote mucus plugging in asthma^26^. The importance of MUC5AC is also highlighted by the finding that MUC5AC knockout mice do not develop airway hyperreactivity or mucus occlusion in response to allergic stimuli. However, MUC5AC transgenic mice do not spontaneously develop airway disease and have normal MCC, suggesting that increased MUC5AC expression alone is not sufficient to cause mucus dysfunction^27–29^.

In this report, we examine the hypothesis that ATP12A promotes the development of dysfunctional mucus during T2 inflammation and thereby impairs MCC. ATP12A is an apically expressed nongastric H^+^/K^+^-ATPase that secretes hydrogen ions (H^+^) in exchange for potassium ions (K^+^) and is thought to be the primary physiologic acidifier of the ASL^30,31^. In CF, decreased HCO_3_^−^ secretion causes the airway surface to become excessively acidic which in turn, increases mucus viscosity and impairs the function of several secreted mediators^10,11,32^. Additionally, the airway inflammation, increased mucus viscosity, and impaired MCC associated with CF airway disease do no develop in CFTR^−/−^ mice unless ATP12A is expressed^33^. Thus, ATP12A appears to be an important mediator of CF airway disease and may contribute to mucus dysfunction in other airway diseases.

## Results

### IL-13 reduces the ASL meniscus volume, inhibits ciliary function, and impairs mucus transport

**Figure 1:**
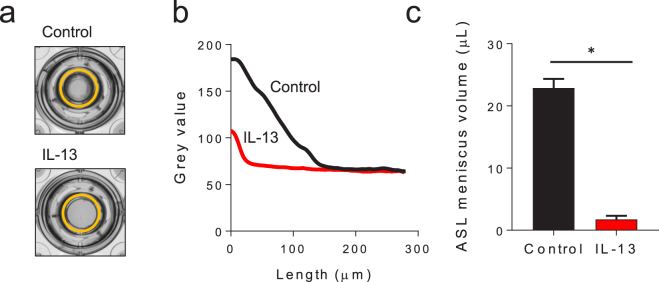
ASL meniscus volume is decreased in primary HBE cell culture following IL-13 exposure. Differentiated HBE cells were cultured ± IL-13 (10 ng/mL) for 3–5 days prior to measuring ASL meniscus volume. (**a**) Representative images of the apical surface of HBE cell cultures with an orange circle outlining the ASL meniscus. (**b**) Representative tracings of the change in light intensity through the apical meniscus used to measure the ASL meniscus volume in HBE cell cultures ± IL-13. (**c**) ASL meniscus volume in HBE cell cultures is decreased by IL-13. Data shown are mean ASL meniscus volume ± SEM of experimental replicates performed on cells from different 12 tissue donors, each replicate with 4–6 cultures, *p < 0.0001 by unpaired Student’s *t*-test. Control donor lines are represented in black, IL-13 treated donor lines are represented in red.

Primary human bronchial epithelial (HBE) cells were cultured on an air-liquid interface and allowed to differentiate. To simulate the effects of T2 inflammation, the differentiated cell cultures were treated with IL-13 (10 ng/mL) for 3–5 days in the basolateral media. As shown in Fig. 1a, a marked change was noted in the appearance of the mucosal surface of the cells following exposure to IL-13. We have previously shown that the airway surface liquid (ASL) forms a meniscus at the interface between the epithelial cell surface and the cell culture transwell filter wall and that this meniscus contains the vast majority of the apical volume. To measure the volume of the ASL contained within the meniscus on HBE cells cultured with IL-13, the cell culture plates were scanned using an optical scanner and the magnitude of the meniscus was measured by analyzing the pattern of light refraction through the fluid^34^. The volume of the ASL meniscus is markedly decreased when HBE cells are exposed to IL-13 as shown in the representative culture images (Fig. 1a, orange outline) and meniscus tracings (Fig. 1b). As shown in Fig. 1c, the ASL meniscus volume was dramatically decreased when the HBE cells were exposed to IL-13 and this was independent of tissue donor disease state (Supplementary Fig. S1). These results indicate that the mucosal airway surface was profoundly altered in the presence of IL-13.

**Figure 2:**
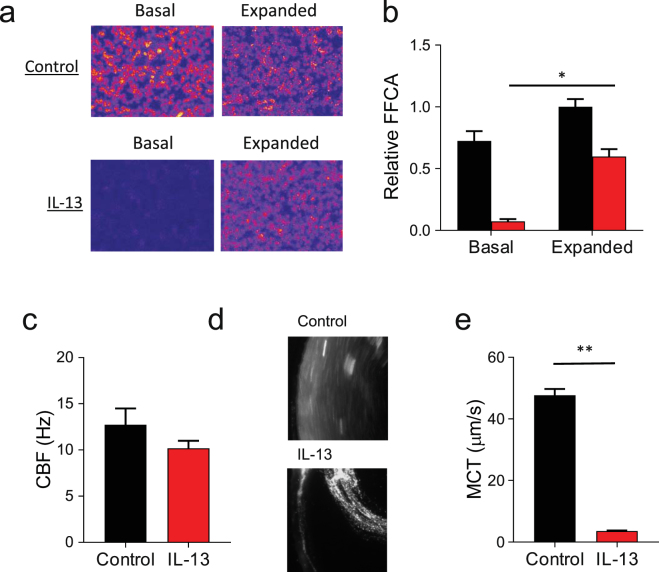
IL-13 impairs mucociliary function in HBE. Control conditions are represented in black, IL-13 treated conditions are represented in red for all plots. (**a**) Combined serial representative images of the fraction of functional ciliary area (FFCA) of HBE cultures ± IL-13. Variations in light intensity representing ciliary motion are indicated in red and yellow. Images were obtained under basal conditions and following the expansion of the ASL with apical rinses of Ringer’s solution. (**b**) Relative FFCA is decreased by IL-13 under basal conditions and improved when the ASL is expanded. Data is expressed as mean FFCA ± SEM relative to control, n = 18 HBE cultures from 3 different tissue donors, *p < 0.0001 via paired Student’s *t*-test. (**b**) Ciliary beat frequency (CBF) in IL-13 treated HBE cell cultures approaches that of untreated controls after expansion with 10 µL Ringer’s solution. Data shown are mean CBF ± SEM (Hz), n = 3 experimental replicates from distinct tissue donors, each replicate is the mean CBF taken from >15 fields. (**c**) Representative images of mucociliary transit in HBE cultures with and without IL-13. (**d**) Mean mucociliary transit (MCT) is decreased by IL-13. Data shown are mean MCT ± SEM (µm/sec), n = 6 cultures each with >5 measured microspheres, **p < 0.0001 via unpaired Student’s *t*-test.

We next assessed whether the IL-13 dependent decreases in ASL volume were sufficient to impair mucociliary transport. To assess ciliary function, we measured the fraction of functional ciliated airway (FFCA), an estimate of the percentage of motile cilia that are able to participate in MCC^35^. A series of sequential images of the HBE cell culture’s apical surface were obtained. As motile cilia will lead to variations in light intensity, the changes of pixel intensity over time directly correlates with regions of increased ciliary motion. As shown in Fig. 2a,b, IL-13 substantially reduced the FFCA compared to control, suggesting that ciliary function is impaired by IL-13. When the apical surface was rinsed and hydrated with several rinses of Ringer’s solution, the FFCA was restored suggesting these changes were not due to direct effects on the cilia. Representative FFCA images are shown in Fig. 2a and the aggregate data of FFCA relative to rinsed control shown in Fig. 2b. As an additional assessment of ciliary function, the ciliary beat frequency (CBF) was measured in HBE following apical rinsing^36^. Although the overall values remained slightly decreased, CBF in IL-13 treated HBE cells approached that of control (Fig. 2c), a finding that is consistent with previous studies of the effect of IL-13 on CBF^26,37^.

Next, to examine whether IL-13 treatment impairs MCC, we measured the rate of rotational mucociliary transport (MCT) in HBE cell cultures. Fluorescent microspheres were added to the apical mucus and the cells were examined 24 h later to allow for the apical fluid bolus to be reabsorbed. An image of the microspheres was then captured with a 5 s exposure. The distance traveled by the microspheres was measured allowing for the calculation of the rotational velocity as previously described^5^. Representative images of the microsphere movement are shown in Fig. 2d. IL-13 exposure dramatically reduced the MCT rate in HBE cell cultures as shown in Fig. 2e. Collectively, these data support the hypothesis that IL-13 impairs ciliary function and mucus transport^26,37^.

### Active ion transport is required for IL-13 dependent ASL meniscus absorption

**Figure 3:**
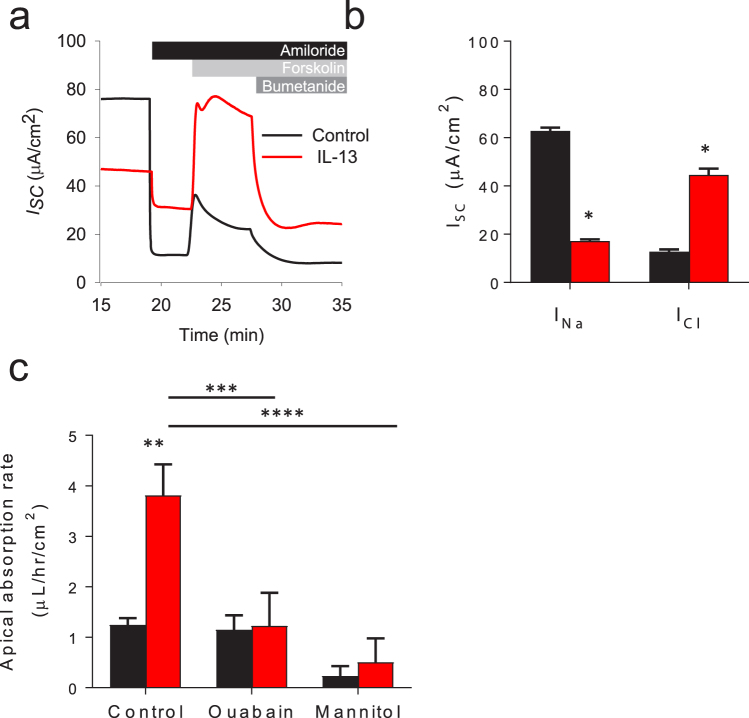
IL-13 dependent ASL meniscus absorption requires active ion transport. (**a**+**b**) Differentiated HBE cells were cultured ± IL-13 for 3 days. The HBE cells were then mounted in Ussing chambers and the amiloride sensitive Na^+^ absorption (*I*_*Na*_) and the forskolin stimulated bumetanide sensitive Cl^−^ secretion (*I*_*Cl*_) was measured. (**a**) Representative *I*_*SC*_ tracings of HBE cell cultures ± IL-13. (**b**) Mean *I*_*Na*_ is decreased and *I*_*Cl*_ is increased by IL-13 in HBE cells. Data shown are mean *I*_*Na*_ and *I*_*Cl*_ ± SEM, n = 4 HBE cell cultures, *p < 0.0001 by unpaired Student’s *t*-test. (**c**) Apical volume absorption rate after the addition of 10 µL Ringer’s solution bolus (control) is increased by IL-13, but is unchanged when ion transport is inhibited by 100 µM basolateral ouabain or through the addition of 10 μL of apical isosmotic mannitol solution. Data shown are mean absorption rate ± SEM (µL/cm^2^/hr), n = 3 tissue donors with 6 experimental replicates per donor, **p = 0.0151, ***p = 0.0453, and ****p = 0.0130 all via unpaired Student’s *t*-test. Control conditions are represented in black, IL-13 treated conditions are represented in red.

The current paradigm of ASL volume homeostasis is that Na^+^ absorption is balanced by Cl^−^ secretion^2^. Published work in primary HBE and animal models of asthma suggest that T2 inflammatory stimuli may promote ASL secretion^20–22^. However, this is inconsistent with both the rheology of asthmatic mucus and the profound decrease in ASL meniscus volume observed in our HBE cell culture model following IL-13 exposure. To reconcile this potential contradiction, we performed a series of experiments to determine whether active ion transport is required for the development of IL-13 mediated reduction in ASL meniscus volume. First, short-circuit experiments were performed to confirm that our cell culture model is consistent with previously published reports of ion transport alterations in IL-13 treated HBE cell cultures. As shown in the representative short-circuit current tracings in Fig. 3a, amiloride was applied to measure the epithelial sodium channel (ENaC) mediated Na^+^ absorption (*I*_*Na*_). Forskolin was then added to stimulate Cl^−^ secretion prior to measuring the bumetanide sensitive Cl^−^ secretion (*I*_*Cl*_). IL-13 treatment substantially reduced electrogenic Na^+^ absorption and increased electrogenic Cl^−^ secretion, as shown in Fig. 3b and in prior reports^20–22^.

Next, we determined whether active ion transport mediates the IL-13 induced changes in the appearance of the HBE mucosal surface. Overall ion transport was blocked through the inhibition of the basolateral Na^+^/K^+^-ATPase with ouabain. A small bolus of Ringer’s solution was then added apically and the ASL meniscus volume was measured over time. As shown in Fig. 3c, absorption of the apical bolus was blocked by basolateral ouabain in HBE cultures with and without IL-13. We then compared absorption rates following the addition of a small bolus of fluid where the physiologic salts had been replaced with non-absorbable isosmotic mannitol. IL-13 treated HBE did not develop hyperabsorption when the ions that contribute to epithelial transport were replaced with mannitol as shown in Fig. 3d. Collectively, these data suggest that active ion transport is required for IL-13 mediated reduction in ASL meniscus volume but that the absorptive pathway is independent of the traditional mechanisms of airway epithelial fluid transport.

### The reduction in the ASL meniscus volume in IL-13 treated cells is due to altered mucus rheology

**Figure 4:**
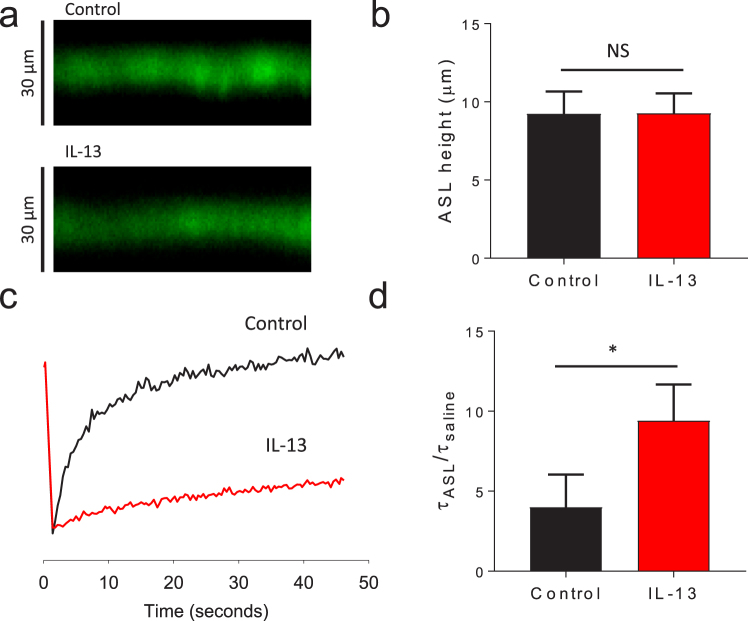
IL-13 increases ASL viscosity. Differentiated HBE cells were cultured ± IL-13 for 3 days. The apical surface was rinsed and labeled with non-absorbable fluorescent dextran. The following day the ASL height was measured and FRAP was performed. (**a**) Representative images of ASL height with and without IL-13. (**b**) The ASL height is not significantly changed by IL-13 in HBE cells as measured by confocal microscopy. Data shown is mean ASL height ± SEM (µm) of experimental replicates from 9 tissue donors, each replicate with 4–6 culture filters, NS by unpaired Student’s *t*-test. (**c**) Representative FRAP tracing demonstrating the relative signal recovery in HBE cell culture with and without IL-13. (**d**) ASL viscosity is increased by IL-13 in HBE cell culture as measured by FRAP. Data shown represents mean τ_ASL_/τ_saline_ ± SEM of replicates from 9 tissue donors, each replicate with 4–6 cultures, *p < 0.0001 by unpaired Student’s *t*-test. Control conditions are represented in black, IL-13 treated conditions are represented in red.

In contrast to the finding that the ASL meniscus volume is depleted by IL-13, prior studies suggest that IL-13 may increase the ASL height of airway epithelia^18,19^. To reconcile this, we measured the ASL height in HBE cell cultures through the addition of a small bolus of non-absorbable fluorescent dextran applied to the apical surface. The ASL height was measured with confocal microscopy the following day to allow for volume reabsorption (representative images shown in Fig. 4a)^38^. The change in ASL height in IL-13 treated cells was highly variable across HBE that were cultured from different tissue donors such that the aggregate data surprisingly did not lead to a change in ASL height as shown in Fig. 4b.

The marked reduction in ASL meniscus volume without a concomitant reduction in ASL height is seemingly contradictory. However, the ASL is a complex fluid comprised of water, mucins, and other macromolecules that determine the ASL’s physical properties^39,40^. Interactions between these surface active components, the apical epithelial membrane, and the transwell filter wall determine how the ASL is partitioned between the thin film and the meniscus. We have previously reported that the overall ASL volume is predominantly partitioned in the meniscus and that changes in the ASL height do not substantially contribute to the measured volume^34^. For example, an increase in ASL height from 10 µm to 20 µm in a transwell filter with a 0.33 cm^2^ surface area (6.5 mm diameter) increases the ASL volume by only 0.33 µL (Volume = height * π * r^2^). Thus, the measured ASL meniscus volume is influenced both by ion transport processes that determine the apical hydration status and by the rheology of the ASL which can alter the partitioning of the ASL across the cell culture surface.

Therefore, we explored the possibility that the reduced volume contained in the meniscus could be explained by an IL-13 dependent alteration in the ASL rheology. The viscosity of a fluid can be inferred by measuring the rate of diffusion of a tracer through the fluid. The ASL was labeled with non-absorbable florescent dextran and the rate of fluorescence recovery after photobleaching (FRAP) was measured the following day^41^. Following photobleaching, the rate of signal recovery was markedly reduced in cells exposed to IL-13, suggesting that the ASL is significantly more viscous in IL-13 treated cells. Representative signal tracings are shown in Fig. 4c and the aggregate data is shown in Fig. 4d. While HCO_3_^−^ and CO_2_ were not present in the basolateral bath or ambient conditions for the reported data, IL-13 also increased the ASL viscosity when the FRAP was measured in cells placed in a HCO_3_^−^ and CO_2_ containing environment (data not shown). This increase in ASL viscosity is consistent with previous reports of the mucus rheology in IL-13 treated airway epithelia and in subjects with asthma^23–25^.

### ATP12A expression is increased by IL-13

**Figure 5:**
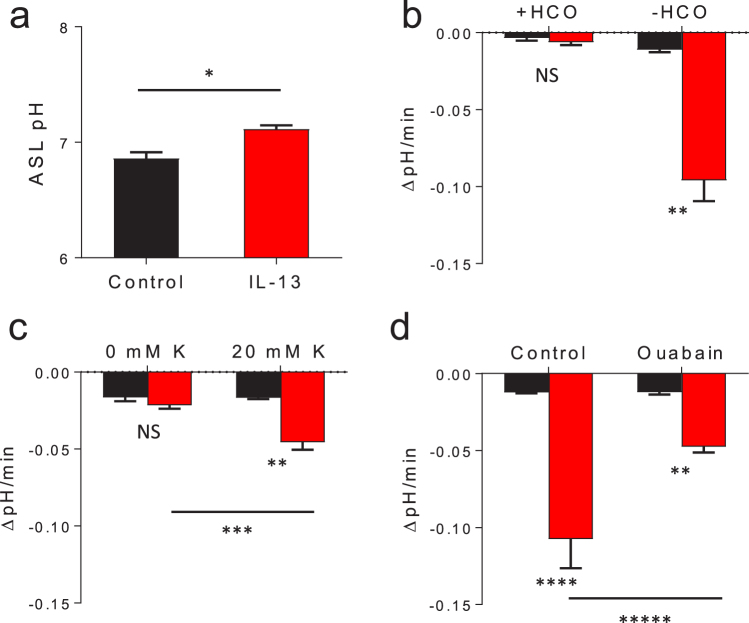
IL-13 increases apical proton secretion. Differentiated HBE cells were cultured ± IL-13, the ASL was labeled with 10 µL BCECF. The cells were incubated in the presence or absence of HCO_3_^−^ as indicated. Control conditions are represented in black, IL-13 treated conditions are represented in red for all plots. (**a**) IL-13 leads to a marginal increase in steady state ASL pH. Following the addition of BCECF, the cells were maintained in solution containing 25 mM HCO_3_^−^ and gassed with 5% CO_2_ at 37 °C. The ASL pH was measured 4 h later. Data shown are mean ASL pH ± IL-13, n = 12 experimental replicates from 3 tissue donors, each replicate with 4 averaged pH readings, *p = 0.0006 by unpaired Student’s *t*-test. (**b**–**d**) A 10 µL bolus of BCECF in an alkaline solution (pH 7.5) was added to the apical surface of HBE ± IL-13. The rate of ASL pH change was measured over 10 min. (**b**) IL-13 increases the ASL acidification rate in HBE cells in the absence of HCO_3_^−^. Data shown represents mean rate of ASL pH change ± SEM (ΔpH/min) from 9 culture filters from 3 tissue donors, **p < 0.0001 by unpaired Student’s *t*-test. (**c**) IL-13 driven proton secretion is potassium dependent. The apical surface was rinsed with 0 mM K^+^ or 20 mM K^+^ prior to pH measurements. Data represents mean rate of ASL pH change ± SEM (ΔpH/min) from 12 replicates from 3 tissue donors, **p < 0.0001 by unpaired Student’s *t*-test and **p = 0.0002 by paired Student’s *t*-test. (**d**) IL-13 mediated increases in apical proton secretion is sensitive to apical ouabain. Rate of ASL pH change was measured in HBE cells cultures ± IL-13 after the addition of a 10 µL bolus of an alkaline solution (pH 7.5). The filters were then rinsed thoroughly and a second 10 µL alkaline bolus with 50 µM apical ouabain was added and the rate of ASL pH change was measured. Data shown represents mean rate of ASL pH change ± SEM (ΔpH/min) from 6 culture filters from 2 tissue donors, ****p = 0.0005 and *p < 0.0001 by unpaired Student’s *t*-test and *****p = 0.0175 by paired Student’s *t*-test.

Evidence suggests that ASL viscosity is influenced by its pH^11^. Therefore, we hypothesized that the increased ASL viscosity observed in IL-13 treated cells may be caused by a reduction in the ASL pH. To measure ASL pH, the ratiometric pH sensitive dye, BCECF, was applied to the apical surface and the fluorescent ratio of 490/440 nm was measured^42^. When the cells were maintained in physiologic conditions (at 37 °C in the presence of HCO_3_^−^ and 5% CO_2_), the ASL was slightly more alkaline in IL-13 treated cells as shown in Fig. 5a. This is not unexpected due to the previously demonstrated increase in expression and activity of pendrin (SLC26A4), an apical, electroneutral, Cl^−^/HCO_3_^−^ exchanging protein^18,19^. We then measured the rate of ASL acidification following the apical addition of alkaline fluids (pH 7.5) with and without bicarbonate (HCO_3_^−^) to control for the effects of HCO_3_^−^ secretion via pendrin. For the HCO_3_^−^ free conditions, the HCO_3_^−^ was replaced with a similar concentration of HEPES buffer (20 mM). As shown in Fig. 5b, the rate of acidification was elevated in IL-13 treated cells in the absence of HCO_3_^−^. This indicates that IL-13 treated cells have an increased capacity to secrete protons in addition to the previously described increases in HCO_3_^−^ secretion.

ATP12A is an electroneutral H^+^/K^+^-ATPase that absorbs extracellular potassium ions (K^+^) in exchange for hydrogen ion (H^+^) secretion. ATP12A is believed to be the primary mechanism for proton secretion in to the ASL, and it has been previously demonstrated that ASL acidification is dependent on ASL K^+^ concentration^30,31^. To assess if the increased rate of acidification mediated by IL-13 was due to ATP12A, we assessed for the rate of ASL acidification in low potassium and high potassium conditions (0 mM K^+^ vs 20 mM K^+^) and, as shown in Fig. 5c, the accelerated rate of proton secretion caused by IL-13 is [K^+^] dependent. In agreement with the hypothesis that IL-13 dependent ASL acidification is mediated by ATP12A, apical ouabain inhibited proton secretion as shown in Fig. 5d. Apical ouabain decreased the ASL viscosity in IL-13 treated cells as shown in Supplementary Fig. S2. These results suggest that ATP12A mediated proton secretion contributes to the development of increased ASL viscosity in IL-13 treated HBE cell culture.

**Figure 6:**
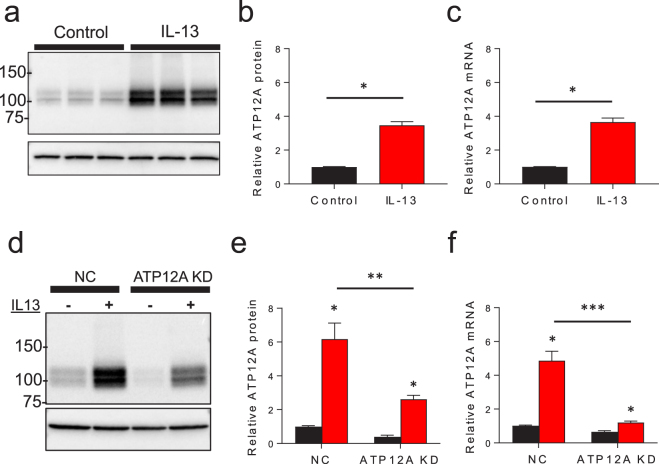
ATP12A expression is increased by IL-13. ATP12A protein and mRNA levels were measured in HBE cells cultured ± IL-13. Control conditions are represented in black, IL-13 treated conditions are represented in red. (**a**) Representative immunoblot of ATP12A protein expression (top) and β-actin (bottom). (**b**) Relative ATP12A protein expression is increased 3.5-fold by IL-13. Data shown is average ATP12A protein level ± SEM as compared to the control ATP12A expression per tissue donor, n = 39 replicates from 13 tissue donors; each replicate consists of 4 pooled culture filters, *p < 0.0001 by Mann Whitney rank sum test. (**c**) IL-13 increases ATP12A mRNA levels. Data shown is mean ATP12A mRNA level ± SEM relative to control levels per tissue donor. N = 84 replicates from 14 tissue donors with three cultures per donor done in duplicate, *p < 0.0001 via Mann Whitney rank sum test. (**d**–**f**) ATP12A lentiviral shRNA knockdown in HBE cultures ± IL-13. HBE cell cultures were transduced with lentivirus expressing shRNA directed toward ATP12A and upon differentiation, relative ATP12A protein and mRNA levels were measured. (**d**) Representative immunoblot of ATP12A protein expression (top) and β-actin (bottom) with and without shRNA ATP12A knockdown ± IL-13. (**e**) Relative ATP12A protein expression in KD cell culture. Data shown is mean relative ATP12A protein expression ± SEM, n = 9 replicate cultures from three tissue donors, *p < 0.0001 via Mann Whitney rank sum and **p = 0.0023 by Student’s unpaired *t*-test. (**f**) Relative ATP12A mRNA levels in KD cell cultures. Data shown is mean relative ATP12A mRNA levels ± SEM, n = 18 replicates from 3 cultures from three tissue donors run in duplicate *p < 0.0001 via Mann Whitney rank sum test and ***p < 0.0001 by Student’s unpaired *t*-test.

To confirm that the IL-13 driven effect on proton secretion and ASL viscosity are due to changes in ATP12A expression, HBE cells were exposed to IL-13 and immunoblotting and rtPCR was performed. ATP12A protein and mRNA expression increased 3–4 fold after being cultured with IL-13. A sample immunoblot is shown in Fig. 6a and the aggregate data across multiple tissue donors is shown in Fig. 6b (protein) and Fig. 6c (mRNA). These results indicate that IL-13 exposure increases ATP12A expression, in agreement with recent microarray data demonstrating that ATP12A expression is increased in HBE cell cultures treated with IL-4, another T2 inflammatory cytokine^43^.

### Il-13 dependent ATP12A expression increases mucus viscosity

**Figure 7:**
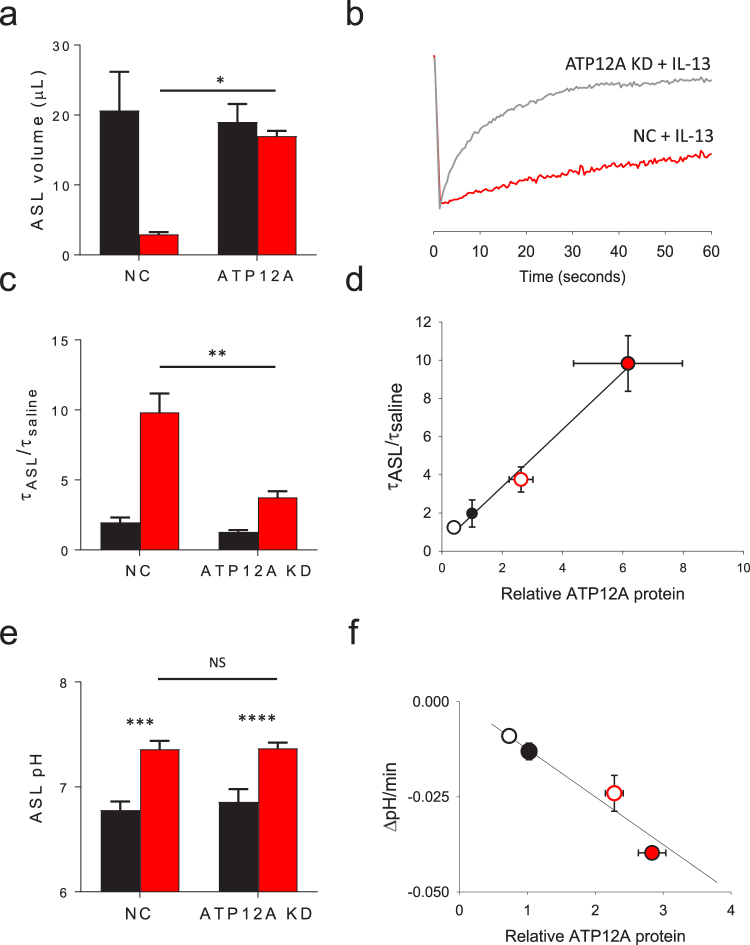
The IL-13 mediated increases in ASL viscosity are ATP12A dependent. Primary HBE cell cultures were transduced with lentivirus expressing shRNA directed towards ATP12A. IL-13 was added to basolateral culture media 3–5 days prior to evaluation. Control conditions are represented in black, IL-13 treated conditions are represented in red. (**a**+**b**) Fluorescent labeled non-absorbable dextran was added to the apical HBE surface. FRAP was measured the following day as a marker of ASL viscosity. (**a**) ASL meniscus volume is unaffected IL-13 when ATP12A is not expressed. Data shown is mean ASL meniscus volume ± SEM. *p < 0.001 by unpaired Student’s *t*-test, n = 3 tissue donors, each with 4–6 experimental replicates. (**b**) Representative FRAP signal recovery tracings for IL-13 treated ATP12A KD and empty vector controls (NC). (**c**) Lack of ATP12A expression is protective against IL-13 mediated increases in ASL viscosity as measured by FRAP. Data shown is average τ_ASL_/τ_saline_ ± SEM in primary HBE cells with and without lentiviral shRNA ATP12A knockdown ± IL-13, n = 12 replicates from three tissue donors, **p = 0.0003 via unpaired Student’s *t*-test. (**d**) The increase in ASL viscosity is directly proportional to the level of ATP12A protein expression. Open circles represent ATP12A knockdown cultures and IL-13 treated cultures are shown in red. Data shown is mean τ_ASL_/τ_saline_ ± SEM (vertical error bars) and mean relative ATP12A protein expression ± SEM (horizontal error bars). Data represents 9 replicates from 3 tissue donors (R^2^ = 0.99, P = 0.0047). (**e**) ASL pH is not changed by lack of ATP12A expression. Data shown is mean ASL pH ± SEM, n = 9 replicates from 2 tissue donors, ***p = 0.0001 and ****p = 0.0014 by unpaired Student’s *t*-test. (**f**) The rate of ASL acidification is proportional to the degree of ATP12A expression. Open circles represent ATP12A knockdown cultures and IL-13 treated cultures are shown in red. Data shown is mean rate of ASL pH change ± SEM (ΔpH/min, vertical error bars) and mean relative ATP12A protein expression ± SEM (horizontal error bars), data represents 6 replicates from 2 tissue donors, R^2^ = 0.74, p < 0.05.

To determine whether ATP12A is required for IL-13’s effect of mucus rheology, a series of experiments were performed to knockdown ATP12A expression. Initially, we identified dicer substrate siRNA that inhibited ATP12A expression (Supplementary Fig. S3a) and this was protective against IL-13 dependent reduction in ASL meniscus volume (Supplementary Fig. S3b). However, as shown in Supplementary Fig. S3c, the knockdown was transient and did not persist to allow for the cells to fully differentiate, limiting our ability to evaluate additional physiological changes at the apical surface. Therefore, we generated lentivirus that expressed ATP12A shRNA to permanently reduce ATP12A expression. As shown in Fig. 6d–f, the transduction of a lentivirus expressing shRNA directed towards ATP12A persistently reduced its expression in primary HBE cells and prevented the IL-13 mediated reduction in ASL meniscus volume (Fig. 7a).

To directly determine whether ATP12A contributes to IL-13 mediated increases in ASL viscosity, FRAP was performed on the ATP12A shRNA knockdown (KD) HBE cells. As shown in a representative FRAP tracing (Fig. 7b) and aggregate data from multiple tissue donors (Fig. 7c), ATP12A KD HBE cells were largely protected from IL-13 induced increases in mucus viscosity. The small increase in ASL viscosity that developed following IL-13 exposure in the ATP12A KD HBE cells is directly proportional to the degree of residual ATP12A expression as shown in Fig. 7d. These results indicate that ATP12A contributes to the development of highly viscous mucus in IL-13 treated HBE and suggest that ATP12A promotes mucus dysfunction during airway T2 inflammation.

As ATP12A has previously been shown to be the primary acidifier of the ASL, we hypothesized that ATP12A KD would increase the ASL pH. Surprisingly, ATP12A KD had no effect on the steady-state ASL pH both in the presence and absence of IL-13 as shown in Fig. 7e. We next measured the effect of ATP12A KD on proton secretion in HCO_3_^−^ free conditions ± IL-13. In contrast to the finding that ATP12A KD did not affect steady state ASL pH, ATP12A KD cultures had reduced IL-13 dependent proton secretion proportional to the degree of residual ATP12A expression (Fig. 7f). These results suggest that while ATP12A functions as a proton pump, the overall contribution to steady state ASL pH is modest.

## Discussion

We have demonstrated that the Type 2 (T2) inflammatory cytokine, IL-13, impairs mucociliary clearance (MCC) by altering the mucus rheology in primary HBE cell culture. IL-13 decreased the airway surface liquid (ASL) meniscus volume with associated impairments in ciliary function and reduction in mucus transport rates. The IL-13 mediated increase in ASL viscosity occurred despite electrophysiological properties that favor ASL secretion and predict a reduction in viscosity^17,20–22^. The primary novel finding of this work is that the increased ASL viscosity seen with IL-13 exposure was mediated through the apical non-gastric proton pump, ATP12A. In support of ATP12A’s pivotal role in IL-13 mediated mucus dysfunction, ATP12A inhibition and knockdown prevented the development of mucus hyperviscosity in IL-13 treated primary cultures of HBE. Therefore, ATP12A appears to be a key mediator of mucus dysfunction in T2 airway disease.

Several lines of evidence support the hypothesis that airway T2 inflammation increases ATP12A expression and thereby promotes mucus dysfunction. IL-13 exposure increased ATP12A expression and function as evidenced by the higher rate of potassium-dependent, ouabain-sensitive proton secretion in IL-13 treated HBE cultures (Figs 5b–d and 6a–c). Recent work by Gorrieri *et al*. demonstrated that IL-4, another T2 inflammatory cytokine, led to increases in ATP12A expression and dramatically reduced the ASL K^+^ concentration, potentially due increased K^+^ absorption through ATP12A^43^. ATP12A expression was highly correlated with ASL viscosity in our experiments. Additionally, ATP12A overexpression modestly increased the ASL viscosity in CFTR^−/−^ mice^33^. Thus, ATP12A appears to be a novel regulator of ASL viscosity and likely contributes to the airway dysfunction that occurs during T2 inflammation.

ATP12A mediates electroneutral hydrogen ion (H^+^) secretion exchange for potassium ions (K^+^) and is believed to function as a primary acidifier of the ASL^30,31^. However, the ASL pH increased following IL-13 exposure regardless of whether ATP12A was expressed (Figs 5a and 7d). These modest effects of ATP12A on the steady state ASL pH were unanticipated, and there are several potential explanations for the lack of correlation between ATP12A expression and ASL pH. First, recent data suggest that bicarbonate readily passes through paracellular pathways in human airway epithelium^44^. The modest ASL acidification rates that we and others have observed in HCO_3_^−^ containing conditions (Fig. 5b) supports the notion that paracellular HCO_3_^−^ permeability is high in airway epithelium^33^. Thus, paracellular HCO_3_^−^ transport is likely to buffer the increased proton secretion though ATP12A in IL-13 treated cells. Additionally, the modest effect of ATP12A on ASL pH following IL-13 exposure can be further explained by the concomitant increase in pendrin (SLC26A4) expression during T2 inflammation^18,19^. As pendrin encodes an apical Cl^−^/HCO_3_^−^ exchanging protein, the ATP12A mediated proton secretion may be masked by coupled HCO_3_^−^ secretion through pendrin. As ASL acidification has recently been demonstrated to increase mucus viscosity^11^, ASL acidification caused by excessive ATP12A mediated proton secretion would appear to be a compelling mechanistic explanation for the mucus dysfunction caused by T2 inflammation. However, our data suggest that the ATP12A effect on mucus rheology is pH independent.

Several pH independent mechanisms to explain how ATP12A increases ASL viscosity were considered. First, the coupled activity of ATP12A and pendrin likely result in net KCl absorption that osmotically drives water absorption and increases the relative mucin content of the ASL. The relatively high concentration of K^+^ in the ASL (20–30 mM) compared to other extracellular fluids suggest that sufficient quantities of potassium ions are present to influence osmotically driven water transport^45^. The notion that KCl transport contributes to ASL regulation is supported by recent work indicating that K^+^ secretion by BK channels is required for airway surface hydration^46–49^. Moreover, significant decreases in the extracellular [K^+^] is predicted to reduce the electrochemical gradient required for Cl^−^ secretion though CFTR and other apical Cl^−^ channels. Thus, the coupled activity of ATP12A and pendrin may promote relative ASL dehydration and thereby increase viscosity and impair mucus clearance. Alternatively, ATP12A activity may increase viscosity through direct mucin modification. The mucin biochemistry and structure may be impacted prior to or during secretion through alterations in the luminal composition or pH of secretory vesicles. Additionally, changes in relative cation concentrations may impact the rate of post-exocytotic swelling experienced by the freshly secreted mucin polymers^50,51^. Airway mucins may also act as a proton buffer and therefore ATP12A driven proton secretion may promote mucus oxidation. Mucus oxidation increases inter- and intra- molecular disulfide bond formation and limits the ability of mucins to hold water^52^. Further studies are required to define the mechanism by which ATP12A increases mucus viscosity.

The airway diseases associated with T2 inflammation and CF are characterized by mucus dysfunction and impaired mucocilliary clearance. However, the pathophysiological mechanisms of these airway diseases appear to be mechanistically different. In CF, alterations in ion transport caused by dysfunctional CFTR render the airway surface vulnerable to a reduction in the ASL height and promote the development of excessively viscous mucus. The ASL height defect has been attributed to decreased Cl^−^ secretion coupled with increased ENaC mediated Na^+^ absorption. The concomitant mucus dysfunction in CF is believed to be due to increased acidity caused by impaired CFTR mediated HCO_3_^−^ secretion. In contrast to the pathobiology of CF, T2 inflammatory cytokines have the opposite effects on ion transport; CFTR and TMEM16A activity are increased and ENaC activity is decreased. We did not appreciate a decrease in ASL height or pH in HBE cells exposed to IL-13 (Figs 4b and 5a). The mucus dysfunction observed in response to T2 inflammation is associated with, and requires, increased ATP12A expression and activity. While ATP12A activity is not abnormal in CF HBE, its expression does appear to be required for the development of the mucus dysfunction and host defense impairments associated with CF airway disease^31,33^. Perhaps the increased exacerbation frequency and increased rate of lung function decline that occurs in individuals with CF and concomitant allergic bronchopulmonary aspergillosis can be explained by additional mucus dysfunction caused by T2 inflammation^53^. Further studies are required to determine whether the mucus dysfunction associated with T2 inflammation is additive to the mucus clearance impairments associated with CF.

In summary, these data indicate that ATP12A is a novel regulator of ASL viscosity and suggest that mucus dysfunction seen in T2 inflammatory airway diseases would be mitigated by the inhibition of ATP12A. The increased mucus viscosity associated with IL-13 appears to be independent of ASL height and develops despite increased electrogenic Cl^−^ secretion and Na^+^ absorption. Interestingly, ATP12A appears to increase ASL viscosity by a pH independent mechanism that will require additional studies to define. Based on the profound effects on mucus transport, ATP12A is a compelling potential therapeutic target to improve mucus dysfunction in T2 airway diseases.

## Materials and Methods

### Primary human airway epithelial cell culture

Following attainment of informed consent, airway segments and lung tissue were obtained from excess pathological tissue following lung transplantation in accordance with a protocol approved by the University of Pittsburgh Investigational Review Board^54^. Epithelial cells were removed from the underlying musculature by blunt dissection, isolated by centrifugation, and re-suspended in BronchiaLife epithelial airway media (Lifeline Cell Technology, Frederick, MD). The cells were grown to 80–90% confluence in collagen-coated flasks and then seeded into collagen-coated 0.33 cm^2^ Costar transwell filters (Corning Costar) at a density of ~5–6 × 10^5^ cells/cm^2^. The cells were cultured at air-liquid interface and considered differentiated when a mucociliary phenotype was apparent on phase contrast microscopy (3–6 weeks). Differentiated HBE cells were cultured ± IL-13 (10 ng/mL added to the basolateral growth media) for 3–5 days prior to being studied.

### ASL meniscus volume measurement

Plates containing 12 culture filter insets were visualized on an optical scanner (Epson V500 optical scanner, Japan). Using an automated image analysis algorithm developed in Image J (NIH, Bethesda, MD), the changes in light intensity as it passes through the apical fluid meniscus were measured along radial spokes from the center to the edge of each transwell filter insert and fit to a sigmoidal function. The area under the curve was integrated to an experimentally determined volume calibration curve using a custom algorithm as previously described^34^. As this algorithm can lead to a negative values for volumes that were too small to be aspirated during the initial volume calibration, the results were analyzed such that the lowest average negative volume per tissue donor was set to zero and this was used as a correction factor for the remaining volume readings for that line.

### Short-circuit current recordings

Differentiated HBE cultures were mounted in Ussing chambers (P2300; Physiological Instruments, San Diego, CA) and cultures were continuously short-circuited with an automatic voltage clamp (VCC MC8, Physiological Instruments) as previously described^54^. Chambers were constantly gassed with a mixture of 95% O_2_/5% CO_2_ at 37 °C, which maintained the pH at 7.4. The apical and basolateral chambers each contained 3 mL of Ringer’s solution (120 mM NaCl, 25 mM NaHCO_3_, 3.3 mM KH_2_PO_4_, 0.8 mM K_2_HPO_4_, 1.2 mM MgCl_2_, 1.2 mM CaCl_2_, and 10 mM glucose). Simultaneous transepithelial resistance was recorded by applying a 10-mV pulse per second via an automated pulse generator. Acquire and Analyze 2.3 (Physiological Instruments) was used to control the voltage clamp and analyze *I*_*SC*_ data.

### ASL height and viscosity measurements

The ASL of rinsed, differentiated, HBE cultures was labeled with 10 µL of 70 kDa FITC-labeled dextran (20 mg/mL, Sigma-Aldrich, St. Louis, MO). The following morning, 50 µL of perfluorocarbon (FC-770, ACROS organics, ThermoFisher, Waltham, MA) was applied to the apical surface to prevent evaporative losses during evaluation. The cultures were then placed on a modified stage of a Nikon TiE inverted microscope equipped with a Nikon confocal A1 scanner and the ASL was visualized with a 40× water immersion objective (Nikon Apo LWD 1.15 NA). To measure the ASL height, a random 320 µm × 20 µm × 30–50 µm region in the center of the culture was selected and imaged. The images of the ASL were analyzed using an automated method as previously described by Song *et al*.^38^.

The ASL viscosity was measured using fluorescent recovery after photobleaching (FRAP) as previously described^41^. After obtaining a baseline image, a small region (6 × 18 µm) in the middle of the ASL was photobleached for 400 milliseconds. Following photobleaching serial images of the region were acquired. The data was fit to an exponential rise to max function to determine the time constant (τ) for fluorescence recovery. The FRAP data is expressed as the ratio of the τ of ASL recovery relative to that of saline (τ_ASL_/ τ_saline_).

### ASL pH measurement

ASL pH was measured using the pH-sensitive ratiometric fluorophore 2′,7′-bis-(2-carboxyethyl)-5-(and-6)-carboxyfluorescein (BCECF) conjugated to 10 kDa dextran (Sigma-Aldrich). 1 mg/mL BCECF dextran was applied to the apical surface. The HCO_3_^−^ containing solution consisted of 120 mM NaCl, 5 mM KCl, 1.2 mM CaCl_2_, 1.2 mM MgCl_2_, 25 mM NaHCO_3_, 5 mM HEPES, and 10 mM glucose and pH measurements were conducted in 5% CO_2_. The HCO_3_^−^ free solution consisted of 125 mM NaCl, 5 mM KCl, 1.2 mM CaCl_2_, 1.2 mM MgCl_2_, 5 mM sodium gluconate, 20 mM HEPES, and 10 mm glucose and the measurements were performed in room air.

The individual cell filter inserts were mounted on a modified stage of a Nikon TiE inverted microscope that maintained the temperature at 37 °C. The ASL was then visualized with a 20× objective (Nikon CFI Apochromat λ 0.75 NA). Ratiometric measurement of the BCECF was performed at 490 nm/440 nm excitation and 535 nm emission wavelengths using a metal arc illuminator and appropriate BCECF filters (Chroma, Bellows Falls, VT). A calibration curve of the BCECF 490 nm/440 nm ratio to ASL pH was generated as previously described^42^. Four predetermined regions on each culture insert were imaged, background corrected, and averaged. Steady state ASL pH was measured after 4 h of equilibration. The rate of ASL pH change was measured over a 10 min period after the addition of the BCECF-study solution.

### Measurements of ciliary function

The fraction of functional ciliated airway (FFCA) measurement was performed as previously described^35^. Briefly, differentiated HBE cell cultures were visualized by phase microscopy on a Nikon TiE inverted microscope. A series of 10 sequential images of the apical surface was obtained and the change in light intensity over time for each pixel was calculated. The area containing motile cilia was derived from the change in pixel intensity above background using a custom algorithm in ImageJ. These measurements were repeated before and after apical rinsing with 10 µL Ringer’s solution.

Ciliary beat frequency (CBF) measurements were performed on differentiated HBE cell cultures. High frame rate videos of the ciliary motion were obtained using a Nikon TiE inverted microscope equipped with an EXi Blue camera (Qimaging, Surrey, Canada). The variation in pixel frequency was fit to a fast Fourier transform (FFT) using a custom algorithm in ImageJ and the mean ciliary beat frequency observed across the field is reported^36^.

Mucociliary transport (MCT) measurement were performed as previously described^5^. Briefly, 1 μm fluorescent microspheres (Invitrogen) were applied to the apical mucus of primary HBE cell cultures. Cultures were visualized on a modified stage of a Nikon TiE inverted microscope and a 5 s exposure was captured. The lengths of the streak created by >5 microspheres per culture were measured.

### Western blotting

HBE cell cultures were rinsed and then lysed by manual scrapping in 100 µL of lysis buffer (10 mM Tris-Cl, 50 mM EGTA, 0.4% sodium deoxycholate, 1% Nonidet P-40, pH 7.4). The resultant proteins were separated by SDS-PAGE on 4–12% gels (Novex Tris/glycine, Invitrogen/ThemoFisher Scientific, Carlsbad, CA) and transferred to nitrocellulose membranes. The membranes were cut between the 50 and 75 kd markers. The membranes from the top half were blocked with 5% nonfat dry milk, blotted with a previously characterized rabbit anti-ATP12A antibody (1:5000, Novus biologicals, Litteton, CO)^33^, and visualized with 1:5000 HRP conjugated anti-rabbit secondary antibodies (BioRad, Hercules, CA). The membranes from the bottom half were blocked with 5% nonfat dry milk, blotted with a monocolonal β actin antibody (Sigma-Aldrich) and visualized with species appropriate HRP conjugated secondary antibodies. Band intensity was quantitated by densitometry using Image J and normalized to level of β-actin expression per individual tissue donor.

### rtPCR

To measure ATP12A mRNA levels, HBE cell cultures were rinsed and then lysed by manual scraping with 200 µL Trizol reagent (Invitrogen) followed by column purification (Zymo, Irvine, CA). The RNA was then converted to cDNA via the High Capacity cDNA RT kiT (Invitrogen). Absolute quantification with real time PCR was performed utilizing the TaqMan method (ATP12A TaqMan gene expression assay Hs01060284_m1, Life Technologies, Carlsbad, CA) with the Lightcycler 96 System (Roche, Switzerland).

### ATP12A knockdown

For ATP12A siRNA knockdown, ATP12A DsiRNA (Integrated DNA Technologies, San Diego, CA) was delivered to primary HBE using reverse transfection as previously described^55^.

Lentiviral vectors containing shRNA directed towards ATP12A (Clone TRCN0000043153, TRC ATP12A shRNA set, GE Dharmacon, Lafayette, CO) were expressed in HEK293 along with the 3^rd^ generation lentiviral packaging constructs pMDLg/pRRE, pRSV-Rev, and pMD2.G (Plasmid #12251, #12253, and #12259 respectively, Addgene, Cambridge, MA). Plasmids were gifts from Didier Trono^56^. Lentiviral particles were created using the ProFection Mammalian Transfection System (Promega, Madison, WI). Undifferentiated HBE cells were transduced with ATP12A shRNA or empty lentiviral vectors when seeded onto transwell inserts as previously described^47,57^. Two to three days later, the selection antibiotic puromycin (1 µg/mL) was added to the basolateral media. The cultures were cultured for 3–4 weeks before experimentation to allow for differentiation.

### Statistical analyses

All data is expressed as mean ± SEM unless otherwise noted. Significance was determined by two-tailed paired or unpaired Student’s *t*-test as noted. Normalized, nonparametric data was analyzed using the Mann-Whitney rank sum test. P < 0.05 was considered statistically significant.

## Electronic supplementary material

Supplementary Information
