# Supplementary material for: ATP12A promotes mucus dysfunction during Type 2 airway inflammation
Source: Sci Rep. 2018 Feb 1;8:2109. doi: 10.1038/s41598-018-20444-8 (PMC5794982; doi:10.1038/s41598-018-20444-8)
Supplement: Supplementary file 1 — Supplementary Information [file 41598_2018_20444_MOESM1_ESM.pdf]

Online Supplemental Information

ATP12A promotes mucus dysfunction during Type 2 airway inflammation

Alison T. Lennox[1], Stefanie L. Coburn[1], John A. Leech[1], Elisa M. Heidrich[1], Thomas R. Kleyman[2,3], Sally E. Wenzel[1], Joseph M. Pilewski[1,3,4], Timothy E. Corcoran[1,5], and Mike M. Myerburg[1,*]

Author Affiliations:

1 – University of Pittsburgh, Department of Medicine, Division of Pulmonary, Allergy, and Critical Care Medicine. UPMC Montefiore Hospital, NW 628, 3459 Fifth Ave, Pittsburgh, PA 15122

2 – University of Pittsburgh, Department of Medicine, Renal-Electrolyte Division; UPMC Montefiore Hospital, NW 628, 3459 Fifth Ave, Pittsburgh, PA 15122

3 - Department of Cell Biology, Department of Pharmacology and Chemical Biology. University of Pittsburgh

4 – Pulmonary Medicine, Allergy, and Immunology, Children's Hospital of Pittsburgh.

5 – Department of Chemical and Petroleum Engineering, Department of Bioengineering. University of Pittsburgh.

Corresponding Author:
Mike M. Myerburg, MD
UPMC Montefiore Hospital, NW628
3459 Fifth Avenue
Pittsburgh, PA 15122
Phone: 412-624-8669
E-mail: myerburgm@upmc.edu

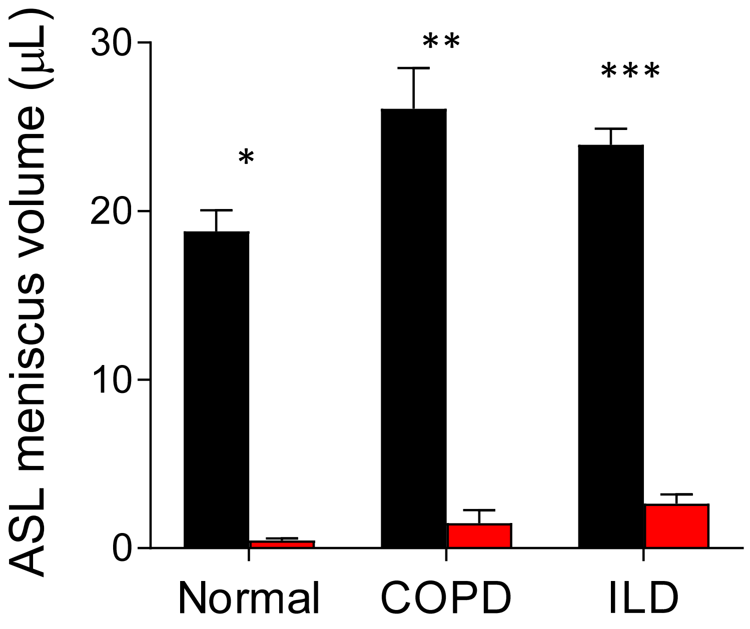

Supplemental Figure S1. Airway surface liquid meniscus volume is decreased by IL13 across multiple disease states. Cultures were obtained from lung transplant recipients with COPD and ILD. 'Normal' lungs were obtained from donor organs that were not deemed suitable for transplant. Data is expressed as mean ASL meniscus volume (µL) ± SEM. Normal: *p=0.005 by unpaired Student's *t*-test, n=2 tissue donors, 4-6 replicates per donor. COPD: **p=0.01 by unpaired Student's *t*-test, n=2 tissue donors, 4-6 replicates per donor. ILD: ***p<0.001 by unpaired Student's *t*-test, n=8 tissue donors, 4-6 replicates per donor. Control conditions are expressed in black and IL-13 treated conditions are in red.

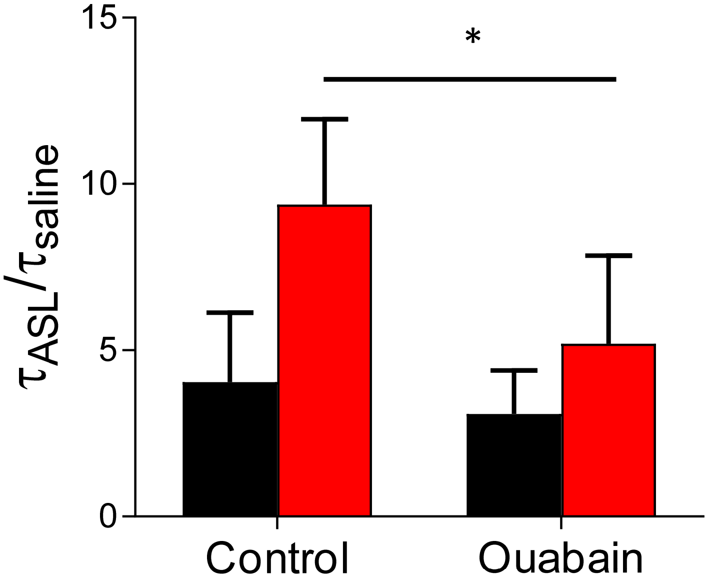

Supplemental Figure S2. Inhibition of ATP12A's function with apical ouabain (50 µM) reduced the IL-13 mediated hyperviscosity as measured by fluorescence recovery after photobleaching (FRAP). Data shown are mean τ_{ASL} relative to τ_{saline} ± SEM, n = 6 tissue donors with 4-6 experimental replicates per donor, *$p<0.05$ by unpaired Student's *t*-test).

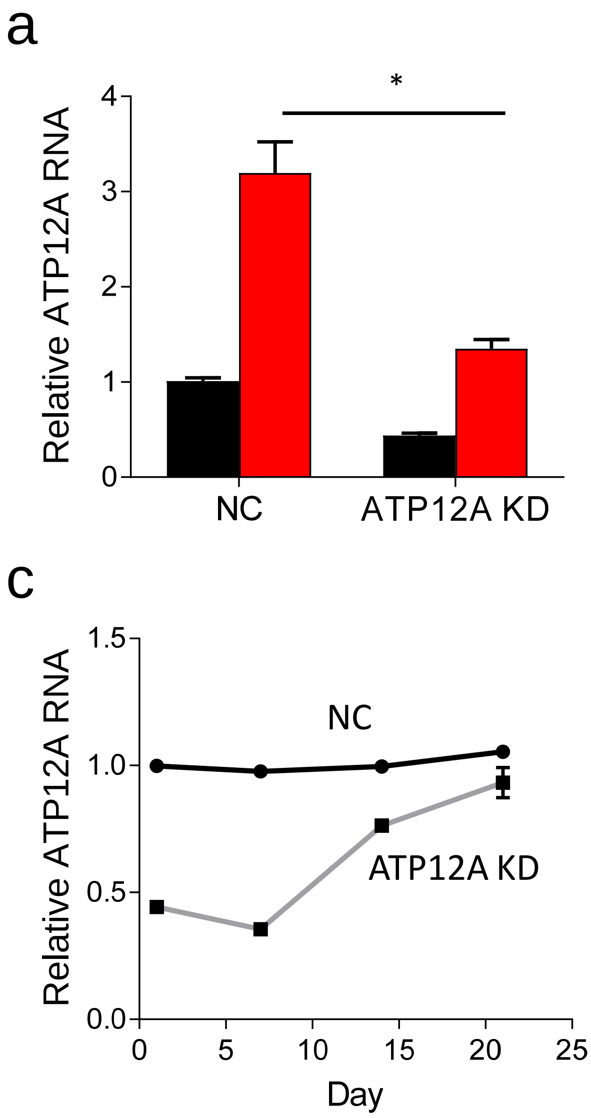

Supplemental Figure S3. ATP12A knockdown by dicer substrate siRNA is protective against IL-13 mediated decreases in ASL meniscus volume. Control conditions are expressed in black and IL-13 treated conditions are in red. (a) Relative ATP12A mRNA with siRNA knockdown ± IL-13. HBE cells were transfected with ATP12A siRNA as they were seeded onto the transwell filter inserts. IL-13 (10 ng/mL) was added to the basolateral growth media on days 4-7 and the cultures were studied on day 7. Data expressed is mean ATP12A mRNA level relative to the untreated NC per individual tissue donor ± SEM. *p=<0.0001 by unpaired Student's t-test, n=24 replicates from 2 tissue donors. (b) ATP12A siRNA knockdown is protective against decreases in ASL meniscus volume. Data expressed is mean ASL meniscus volume (μL) ± SEM **p<0.0001 by unpaired Student's t-test, n=12 experimental replicates from 2 tissue donors. (c) ATP12A siRNA KD was most pronounced in the first week of ALI culture and did not persist until full cellular differentiation (average 21 days). Data shown is mean relative ATP12A RNA level with and without siRNA KD ± SEM over time, n=4 experimental replicates per time point from an individual tissue donor.
